# Weak Organic Acids Decrease *Borrelia burgdorferi* Cytoplasmic pH, Eliciting an Acid Stress Response and Impacting RpoN- and RpoS-Dependent Gene Expression

**DOI:** 10.3389/fmicb.2017.01734

**Published:** 2017-09-29

**Authors:** Daniel P. Dulebohn, Crystal L. Richards, Hua Su, Kevin A. Lawrence, Frank C. Gherardini

**Affiliations:** Laboratory of Zoonotic Pathogens, Gene Regulation Section, Division of Intramural Research, Rocky Mountain Laboratories, National Institute of Allergy and Infectious Diseases, National Institutes of Health, Hamilton, MT, United States

**Keywords:** Lyme disease, gene regulation, arthropod vector, acid stress response, virulence factor expression

## Abstract

The spirochete *Borrelia burgdorferi* survives in its tick vector, *Ixodes scapularis*, or within various hosts. To transition between and survive in these distinct niches, *B. burgdorferi* changes its gene expression in response to environmental cues, both biochemical and physiological. Exposure of *B. burgdorferi* to weak monocarboxylic organic acids, including those detected in the blood meal of fed ticks, decreased the cytoplasmic pH of *B. burgdorferi in vitro*. A decrease in the cytoplasmic pH induced the expression of genes encoding enzymes that have been shown to restore pH homeostasis in other bacteria. These include putative coupled proton/cation exchangers, a putative Na^+^/H^+^ antiporter, a neutralizing buffer transporter, an amino acid deaminase and a proton exporting vacuolar-type V_o_V_1_ ATPase. Data presented in this report suggested that the acid stress response triggered the expression of RpoN- and RpoS-dependent genes including important virulence factors such as outer surface protein C (OspC), BBA66, and some BosR (*Borrelia* oxidative stress regulator)-dependent genes. Because the expression of virulence factors, like OspC, are so tightly connected by RpoS to general cellular stress responses and cell physiology, it is difficult to separate transmission-promoting conditions in what is clearly a multifactorial and complex regulatory web.

## Introduction

Pathogens disseminated by arthropod vectors are responsible for many emerging and reemerging infectious diseases that impose a significant burden on public health worldwide. Successful vector-borne transmission requires the pathogen to survive and replicate in very dissimilar hosts. Typically, bacterial pathogens sense, respond, and adapt to host environments during vector–host–vector (infectious cycle) transitions. These adaptations ensure that the appropriate changes in gene expression occur at the time suitable for successful colonization of these habitable hosts. Identifying the environmental and biochemical changes that occur in these distinct niches and understanding how pathogens respond during the infectious cycle can provide valuable insight into the pathogenesis of vector-borne pathogens.

The hard tick *Ixodes scapularis* can transmit pathogenic protozoans, viruses, and bacteria, including the causative agent of Lyme disease, the spirochete, *Borrelia burgdorferi* (Burgdorfer et al., 1982; Steere et al., 1983; Nelder et al., 2016). *B. burgdorferi* survives strictly in a vector- or host-environment (Lane et al., 1991) and successfully transitions between these to complete its infectious cycle. Transmission from ticks and establishment of infection in a host requires an atypical signaling cascade, the Rrp2–RpoN–RpoS cascade, to direct key changes in gene expression (Yang et al., 2003; Fisher et al., 2005; Dunham-Ems et al., 2012). This signaling cascade consists of the cytoplasmic response regulator protein-2 (Rrp2) and two alternative sigma factors, RpoN and RpoS. Activation of Rrp2 promotes RpoN-directed transcription of *rpoS* and subsequent RpoS-directed transcription of genes encoding proteins required for survival and key virulence factors (Hübner et al., 2001; Clifton et al., 2006; Burtnick et al., 2007; Caimano et al., 2007; Samuels, 2011).

Multiple environmental parameters fluctuate during the infectious cycle and changes in pH (7.5 → 6.8), temperature (25° → 34°), osmolarity (600 mOsm → 300 mOsm), nutrient availability and cell density (∼1–2 × 10^8^ cells/mL) have been shown to trigger the expression of RpoS-dependent virulence factors *in vitro* (Schwan et al., 1995; Carroll et al., 1999; Yang et al., 2000; Bontemps-Gallo et al., 2016). Additionally, cells encounter reactive nitrogen species (RNS) and reactive oxygen species (ROS) during the infectious cycle (Bourret et al., 2016). These stressors can promote changes in gene expression (Hyde et al., 2006) and require protective stress response proteins (e.g., UvrB, MutS; Bourret et al., 2016; Ramsey et al., 2017), some directly regulated by the *Borrelia* oxidative stress regulator, BosR (e.g., SOD, CoADR), for survival (Boylan et al., 2006; Esteve-Gassent et al., 2009; Eggers et al., 2011). Moreover, acetate (Xu et al., 2010; Van Laar et al., 2012; Richards et al., 2015) and benzoate (Richards et al., 2015) activate RpoS expression, utilizing a mechanism that is independent of the acetate-derived acetyl-phosphate (Ac-P) phosphorylation of Rrp2 (Richards et al., 2015). How weak monocarboxylic acids affect the physiology and gene expression in *B. burgdorferi* is unclear.

Membrane-permeable weak monocarboxylic organic acids, like acetate and benzoate, have been shown to trigger acid/proton stress in some bacteria (Kirkpatrick et al., 2001). This stress occurs when excess, free protons accumulate in the cytoplasm (Foster, 1999). Protonated weak acids can diffuse across the cell membrane, dissociate in the cytoplasm, and increase the concentration of intracellular protons. This generates differences in the pH between the external environment (pH_e_) and the cytoplasm (pH_i_), acidifies the cytosol, decreasing the activity of pH-sensitive enzymes, and disrupting the proton motive force (PMF) (Bearson et al., 1997; Foster, 1999, 2004; Kanjee and Houry, 2013). To survive, many bacteria initiate an acid stress response to restore pH homeostasis. Disparate mechanisms are employed to reestablish a favorable pH_i_ including: (i) coupled proton exchange with cations or anions (antiporters); (ii) generating or transporting neutralizing buffering molecules including proteins (ionizable side groups can act as cytosolic buffers), polyamines, polyphosphates, and inorganic phosphates; (iii) utilizing amino acid decarboxylases and/or deaminases; (iv) the synthesis of acid resistant fatty acids and lipids; and (v) reversing proton import via the F_1_/F_o_ ATPase by hydrolyzing ATP to exclude protons (Bearson et al., 1997; Foster, 1999; Slonczewski et al., 2009). Many of the systems utilized by other bacteria to neutralize excess protons in the cytoplasm are absent in *B. burgdorferi*, including amino acid synthesis, amino acid decarboxylases, fatty acid biosynthesis, most neutralizing metabolites, an F_1_/F_o_ ATPase and some proton pumping transporters (Fraser et al., 1997). Lacking a respiratory chain or cytochrome system for eliminating protons from the cytoplasm, *B. burgdorferi* utilizes a proton pumping V_o_V_1_ vacuolar-type ATPase that hydrolyzes ATP and pumps protons to maintain the essential PMF (Fraser et al., 1997; Radolf and Samuels, 2010). The paucity of recognizable mechanisms to deal with perturbations of cytoplasmic pH suggests that *B. burgdorferi* is not exposed to significant levels of acids (HCl) or weak monocarboxylic organic acids or is using an atypical mechanism(s) to deal with acidification of its cytosol. However, the molecular mechanism and physiological significance of the acetate/benzoate stimulated changes in gene expression was previously unclear (Xu et al., 2010; Richards et al., 2015).

To understand the molecular mechanism and biological significance of the acetate-induced changes in gene expression and determine if this induction is related to a typical acid stress response, we analyzed the effect of membrane-permeable acids on *B. burgdorferi*. We demonstrated that membrane-permeable acids decrease pH_i_ and exposure to the membrane permeable acid propionate activates a previously uncharacterized acid stress response. Exposure to monocarboxylic organic acids triggered an increase in the transcription of genes encoding proteins that represent a unique approach to relieving acid stress. Moreover, our data established that membrane-permeable acids are present during the infectious cycle in both mouse and rabbit sera, and in tick midgut contents following a blood meal. These monocarboxylic acids may act as environmental cues during the infectious cycle to trigger changes in gene expression to generate protective responses to multiple stressors and likely promote long-term survival of *B. burgdorferi* throughout the infectious cycle.

## Results

### Membrane-Permeable Acids Decrease Cytoplasmic pH in *B. burgdorferi*

The addition of acetate to *B. burgdorferi* growth medium promotes the expression of RpoS and RpoS-dependent genes (e.g., OspC, DbpA, etc.; Xu et al., 2010; Van Laar et al., 2012; Richards et al., 2015). The acetate-triggered induction was presumed to be a result of increased levels of intracellular Ac-P, which, in turn, initiated the Rrp2–RpoN–RpoS regulator cascade (Xu et al., 2010). However, a recent study has shown that acetate can upregulate RpoN/RpoS-dependent proteins in the absence of Ac-P (Richards et al., 2015). The molecular mechanism and physiological significance of the acetate-triggered induction of virulence factors has remained unclear.

**FIGURE 1:**
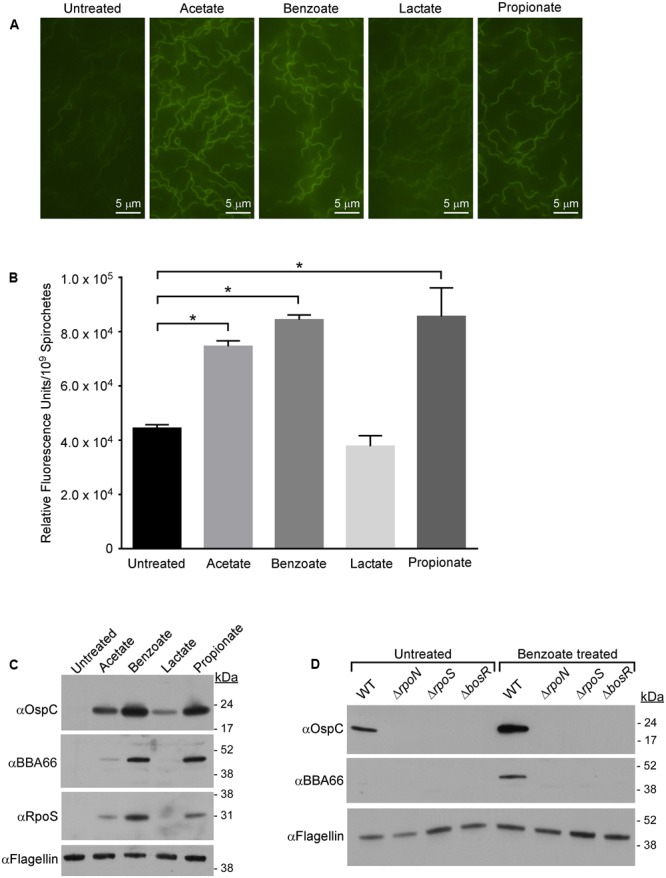
Membrane-permeable acids decrease pH_i_ and promote RpoS-dependent virulence factor expression. **(A)** Fluorescence microscopy of *B. burgdorferi* strain B31-A3 grown in media alone (untreated) or in the presence of 20 mM acetate, benzoate, lactate, or propionate and stained with the membrane-permeable pH-sensitive dye pHrodo Green. **(B)** Quantitative microplate assay analysis of the relative fluorescence levels of spirochetes grown in media alone (untreated) or in the presence of acetate, benzoate, lactate, or propionate. Equivalent numbers of spirochetes (1 × 10^9^) were used for each condition tested. The pHrodo Green dye increases in fluorescence in acidic environments, the bar represents the mean determined from three independent biological replicates and the standard deviation is displayed with brackets. Asterisks denote a statistically significant difference in the mean determined using a one-way ANOVA where *p* < 0.05. **(C)** Immunoblot of RpoS and RpoS-dependent proteins OspC and BBA66 in B31-A3 in response to growth in BSK-II medium containing acetate, benzoate, lactate, or propionate. **(D)** Immunoblot of OspC, BBA66 in *B. burgdorferi* wild-type (WT), B31-A3Δ*rpoN* (Δ*rpoN*), B31-A3Δ*rpoS* (Δ*rpoS*), B31-A3Δ*bosR* (Δ*bosR*) strains grown in the absence or presence of benzoate. Flagellin is included as a loading control and positions of molecular weight standards are indicated on the right in kDa. All experiments were performed with at least three biological replicates and representative immunoblots are presented in **(C,D)**. Fields for images in **(A)** were selected at random from micrographs found in Supplementary Figure S2.

One consequence of exposing bacterial cells to membrane permeable acids, like acetate, is a drop in pH_i_ (acid stress) which can retard growth and, under extreme conditions, cause cell death (Foster, 1999; Wilks and Slonczewski, 2007; Slonczewski et al., 2009; Wilks et al., 2009). Since acetate-induced expression of OspC and RpoS was independent of Ac-P concentration, it seemed likely that this “acetate effect” might result from acetate directly decreasing pH_i_. To test this possibility, we monitored the cytoplasmic pH of *B. burgdorferi* exposed to various membrane-permeable acids using the pH-responsive membrane-permeable dye pHrodo Green (effective pH range 4–7) (Miksa et al., 2009). *B. burgdorferi* B31 strain A3 spirochetes were grown in BSK-II media containing acetate, benzoate, lactate, or propionate, stained with pHrodo Green and visualized using fluorescence microscopy (**Figure 1A**). Spirochetes grown in BSK-II alone and exposed to pHrodo Green showed little fluorescence compared to background indicating a near neutral intracellular pH (**Figure 1A**, untreated). However, spirochetes grown in media containing acetate, benzoate, or propionate and exposed to the dye were demonstrably brighter, suggesting that these acids had acidified the cytosol. Cells exposed to lactate showed little change in fluorescence, indicating that this acid had a minor effect on intracellular pH (**Figure 1A**, lactate). We confirmed the fluorescence microscopy data using a more sensitive quantitative fluorescence microplate assay as described in “Materials and Methods” (**Figure 1B**). Cells exposed to acetate, benzoate, or propionate had a statistically significant increase in fluorescence compared to the untreated control (**Figure 1B**, untreated, acetate, benzoate, propionate) while lactate (**Figure 1B**, lactate) showed no change in fluorescence relative to the control. Although fluorescence microscopy showed a slight change in fluorescence for cells exposed to lactate, there was no change in the microplate assay, suggesting that subtle changes in fluorescence are difficult to quantify using this assay. Additionally, the pH of the buffered media did not change throughout the experiment. The result for lactate was predictable as *B. burgdorferi* is a homofermenter whose sole end-product of central metabolism is lactate. Thus, these bacteria have a highly efficient lactate secretion system that would actively excrete lactate as fast as it could enter the cell. These data suggest that membrane permeable acids can directly affect the pH_i_ of *B. burgdorferi.*

### A Decrease in pH_i_ Promotes RpoN- and RpoS-Dependent Virulence Factor Expression

Having demonstrated that membrane-permeable weak acids decrease pH_i_, we next sought to determine if the induction of RpoS and OspC in the presence of acetate (Xu et al., 2010; Van Laar et al., 2012; Richards et al., 2015) is due to an acid stress response in *B. burgdorferi*. We analyzed the effect of acetate, benzoate, lactate, and propionate on RpoS and RpoS-dependent virulence factors OspC and BBA66 during mid-logarithmic phase (1–5 × 10^7^ spirochetes/mL) to avoid growth-dependent (“stationary phase”) induction of RpoS (Yang et al., 2000). Cell lysates of spirochetes grown in BSK-II containing acetate, benzoate, lactate, or propionate were analyzed by immunoblot for production of RpoS, OspC, and BBA66. Benzoate and propionate had the greatest effect on RpoS expression, causing a marked increase in RpoS expression compared to the untreated control (**Figure 1C**). Acetate led to a moderate increase in RpoS levels while lactate did not increase RpoS expression. Benzoate and propionate, followed by acetate, resulted in the greatest increase in expression of the RpoS-dependent factors OspC and BBA66 (**Figure 1C** and Supplementary Figure S1). Lactate increased the expression of only OspC compared to the untreated control (**Figure 1C** and Supplementary Figure S1). Taken together, these data suggest that a decrease in pH_i_ promotes the expression of RpoS and RpoS-dependent virulence factors, with non-metabolizable acids having the greatest affect on both RpoS production and pH_i_.

Synthesis of RpoS and RpoS-dependent virulence factors requires activation of the Rrp2–RpoN–RpoS signal transduction cascade (Yang et al., 2003; Fisher et al., 2005; Burtnick et al., 2007) and the transcriptional regulator BosR (Ouyang et al., 2009). To determine if acid stress promotes production of OspC and BBA66 through the Rrp2–RpoN–RpoS signaling pathway, *B. burgdorferi* strains B31-A3 (wild-type, WT), B31-A3Δ*rpoN* (Δ*rpoN*), B31-A3Δ*rpoS* (Δ*rpoS*), and B31-A3Δ*bosR* (Δ*bosR*) were grown in medium containing benzoate and we determined the level of OspC and BBA66 by immunoblot (**Figure 1D**). Unlike the WT strain, in the Δ*rpoN*, Δ*rpoS*, and Δ*bosR* mutant strains, production of OspC and BBA66 were abrogated (**Figure 1D**). Therefore, in response to membrane permeable acids, synthesis of RpoS and RpoS-dependent virulence factors likely proceed through the Rrp2–RpoN–RpoS pathway and this response requires BosR. These results suggest a generalized response to membrane-permeable acids where a decrease in pH_i_ activates the Rrp2–RpoN–RpoS/BosR signaling cascade resulting in virulence factor synthesis in *B. burgdorferi*.

### Membrane-Permeable Acids Activate the Rrp2–RpoN–RpoS Signaling Cascade and the Oxidative Stress Regulon

**FIGURE 2:**
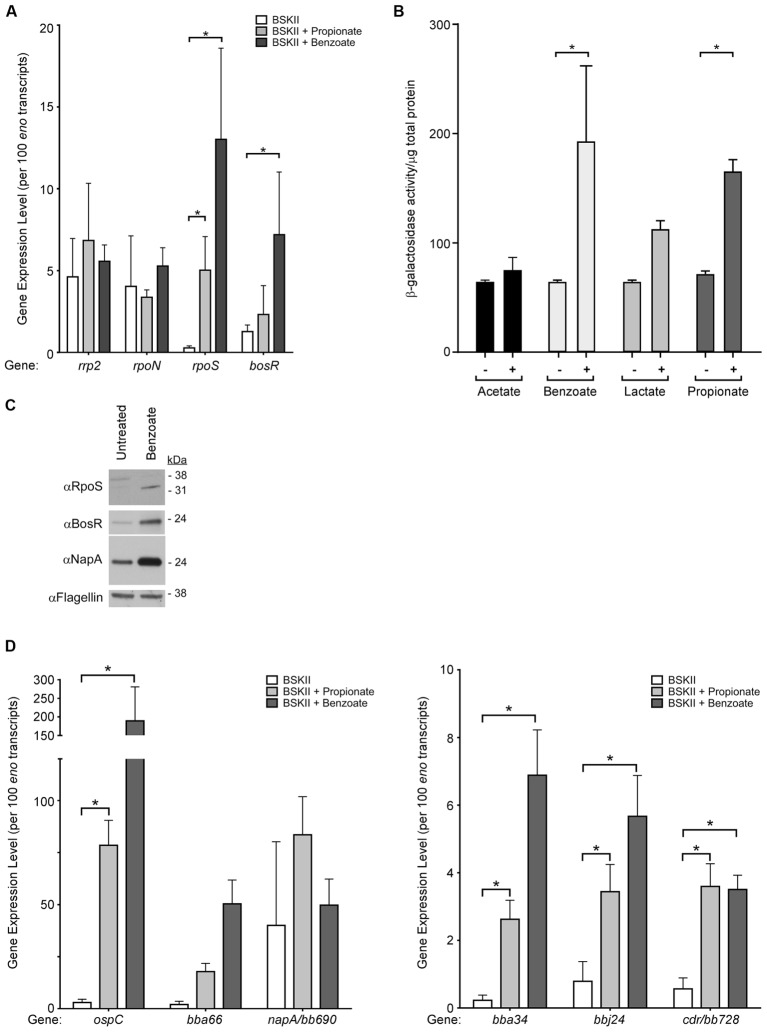
Membrane-permeable acids activate transcription of RpoS-dependent virulence factors and the oxidative stress regulon. Gene expression and immunoblot analysis of *B. burgdorferi* strain B31-A3 in response to membrane-permeable acids. **(A)** qRT-PCR analysis of key regulators of the Rrp2–RpoN–RpoS/BosR signal transduction cascade, *rrp2, rpoN, rpoS*, and *bosR*. **(B)** *B. burgdorferi* strain B31-A3/pBSV2G-*rpoS*p-*lacZ*_Bb_ containing a shuttle vector with the *rpoS* promoter fused to *lacZ*_Bb_ was grown in the (–) absence or (+) presence of acetate, benzoate, lactate, or propionate and β-galactosidase activity in cell lysates was analyzed. Brackets with asterisks indicate a statistically significant difference between the means with *p*-values <0.05 determined using a two-way ANOVA. **(C)** Immunoblot analysis of RpoS, BosR, and the BosR-regulated factor NapA/BB690 in *B. burgdorferi* in response to benzoate. Flagellin is included as a loading control and the position of molecular weight standards is indicated on the right in kDa. **(D)** qRT-PCR analysis of RpoS-dependent genes *ospC, bba66, bba34, bbj24* and BosR-regulated genes *napA/bb690* and *cdr/bb728*. Expression levels were normalized to the *eno* gene encoding enolase and are represented as transcript copies per 100 *eno* copies. Asterisks denote a statistically significant difference of the mean determined using a two-way ANOVA with *p* < 0.05. All experiments were performed using at least three biological replicates with the mean and standard deviation displayed in **(A,B,D)** and a representative immunoblot presented in **(C)**.

Regulation of the Rrp2–RpoN–RpoS cascade in *B. burgdorferi* is complex, with multiple components regulated transcriptionally, post-transcriptionally, or post-translationally (Yang et al., 2003; Fisher et al., 2005; Burtnick et al., 2007; Lybecker and Samuels, 2007; Dulebohn et al., 2014; Richards et al., 2015). Having established that RpoS increases in response to acid stress, we next sought to determine if *rpoS*, or other key components of the Rrp2–RpoN–RpoS cascade, are transcriptionally regulated in response to membrane permeable acids. Using quantitative reverse transcriptase PCR (qRT-PCR) we analyzed the level of *rrp2* and *rpoN* in response to membrane permeable acids and found they were relatively unchanged under acid stress conditions (**Figure 2A**). Conversely, *rpoS* transcript levels demonstrated a statistically significant increase under the same conditions, suggestive of transcriptional regulation of *rpoS* under acid stress and consistent with the immunoblot data demonstrating an increase in RpoS (**Figures 1C, 2C**). Additionally, *B. burgdorferi* strain B31-A3 harboring a plasmid containing the *rpoS* promoter fused to the *lacZ*_Bb_ gene (Hayes et al., 2010) exhibited a significant increase in β-galactosidase activity in the presence of benzoate or propionate (**Figure 2B**).

The level of the *rpoS* transcript expressed in *B. burgdorferi* is intricately linked to the BosR (Ouyang et al., 2009) and BosR is required for expression of OspC and BBA66 in response to acid stress (**Figure 1D**). We quantified the level of *bosR* transcript and found that expression remained relatively unchanged in the presence of propionate, but increased significantly in spirochetes exposed to benzoate (**Figure 2A**). Having observed a statistically significant increase in *bosR* transcript levels in response to benzoate we further evaluated BosR and the BosR-regulated protein NapA/BB0690 by immunoblot. In response to benzoate, both BosR and NapA levels increased compared to the untreated control (**Figure 2C**). Taken together, these data suggest that *rpoS* and *bosR* transcript levels increase in response to acid stress, leading to increased levels of these key regulators that may affect expression of both RpoS-dependent and BosR-regulated genes, suggestive of a possible overlap between an acid stress response and the oxidative stress response in *B. burgdorferi*.

Since RpoS and BosR levels increased under acid stress, we quantified RpoS-dependent and BosR-regulated transcripts in response to acid stress by qRT-PCR. These included the RpoS-dependent transcripts *ospC, bba66, bbj24, bba34* and the BosR-regulated genes *napA*/*bb690* and *cdr*/*bb728* (**Figure 2D**). *B. burgdorferi* was grown in BSK-II supplemented with benzoate or propionate and transcript levels indicated that the RpoS-dependent genes *ospC, bba66, bbj24*, and *bba34*, were induced in response to propionate or benzoate compared to the untreated control (**Figure 2D**). Consistent with these data, β-galactosidase activity was significantly increased in spirochetes containing a plasmid with the *lacZ*_Bb_ gene fused to the *ospC* promoter in the presence of acetate, benzoate, or propionate (Supplementary Figure S3). Transcript levels of the BosR-regulated gene (Boylan et al., 2003; Hyde et al., 2006, 2009; Ouyang et al., 2009) *napA*/*bb690* remained relatively unchanged, however, the level of *cdr/bb728*, encoding CoA reductase, was significantly increased in response to both propionate and benzoate (**Figure 2D**). These data indicate that RpoS-dependent and a BosR-dependent genes are induced in response to acid stress, also suggestive of an overlap between an acid stress response and the oxidative stress response in *B. burgdorferi*.

### Propionate Promotes Transcription of Genes Typically Associated with Protection from Acid Stress

In response to acid stress, many bacteria activate an acid stress response (Marquis et al., 1987; Bearson et al., 1997; Foster, 1999, 2004; Slonczewski et al., 2009; Kanjee and Houry, 2013) to stabilize pH_i_ and retain the PMF (Bearson et al., 1997; Slonczewski et al., 2009; Wilks et al., 2009). *B. burgdorferi* is thought to lack “typical” bacterial stress responses (Caimano et al., 2004) and is missing many of the enzymes and transporters characteristically utilized to mitigate acid stress (Fraser et al., 1997). However, the genome does encode a vacuolar-type V_o_V_1_ ATPase, an arginine deiminase system (ArcA-ArcB-ArcD), and several proton/ion pumping transporters/symporters that could play a role in maintaining pH_i_ and the PMF in response to acid stress. To determine if *B. burgdorferi* responds to acid stress by increasing the transcription of genes encoding protective enzymes or transporters associated with an acid stress response we monitored select genes in response to spirochete growth under acid stress conditions. To generate acid stress conditions, we exposed spirochetes to propionate, as it activates the Rrp2–RpoN–RpoS cascade (**Figure 1C**) and unlike lactate and acetate, is not actively exported from *Borrelia* or utilized in undecaprenyl-P synthesis (Fraser et al., 1997). Unable to be metabolized like acetate or exported like lactate, propionate may accumulate in the cytoplasm, enhancing its ability to decrease pH_i_. Additionally, the tick microbiome does not harbor bacteria predicted to generate benzoate, however, several species of bacteria found in the *I. scapularis* midgut could potentially produce propionate as a metabolic end-product, likely exposing *B. burgdorferi* to this acid during the infectious cycle (Narasimhan et al., 2014).

**FIGURE 3:**
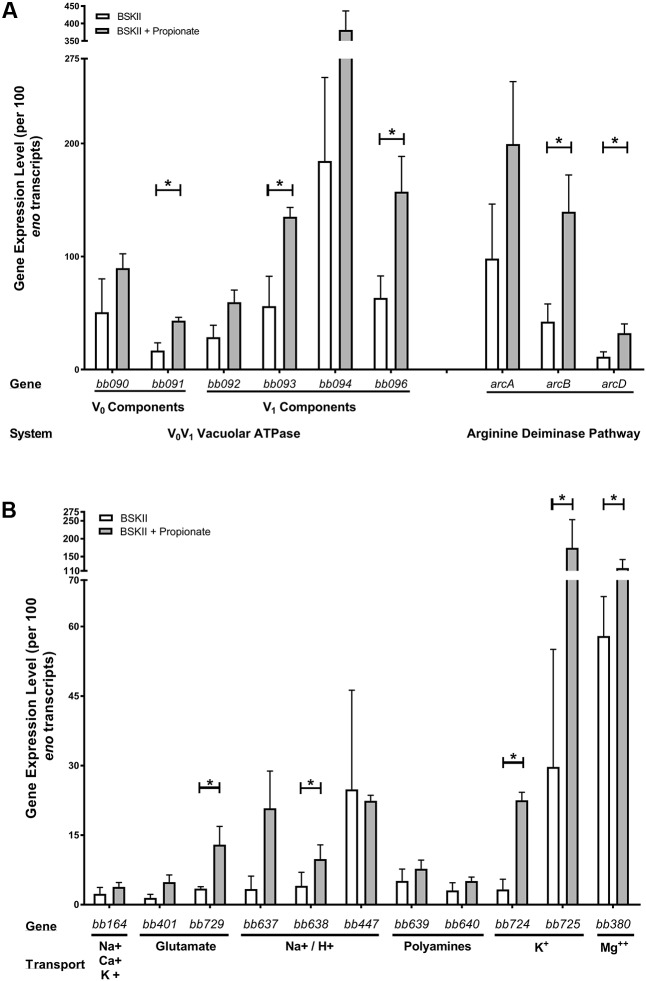
Membrane-permeable acids promote the expression of genes that encode proteins involved in protection from acid stress. qRT-PCR of select genes following growth of spirochetes in BSK-II medium or BSK-II medium containing propionate. Transcript levels of the **(A)** proton pumping vacuolar-type V_o_V_1_ ATPase (*bb090, bb091, bb092, bb093, bb094, bb096*), components of the arginine deiminase pathway (*arcA*/*bb841, arcB*/*bb842, arcD*/*bb843*) and **(B)** H^+^ or metabolite transporters or symporters for Na^+^, Ca^++^, K^+^ (*bb164*), glutamic acid (*bb401, bb729*), Na^+^/H^+^ (*bb637, bb638, bb447*), polyamines (*bb639, bb640*), K^+^ (*bb724, bb725*), and Mg^++^ (*bb380*) using gene specific primers and probes. Expression levels were normalized to the *eno* gene and the mean from three independent biological replicates are represented as transcript copies per 100 *eno* copies along with the standard deviation denoted by brackets. Asterisks denote a statistically significant difference of the mean determined by Student’s unpaired *t*-test where *p* < 0.05.

The *Borrelia* V_o_V_1_ ATPase (Fraser et al., 1997) is hypothesized to hydrolyze ATP to translocate protons out of the cytoplasm to maintain the PMF and drive proton-coupled transport (Radolf and Samuels, 2010). This is uncommon in bacteria but vacuolar V_o_V_1_ ATPases are often utilized by eukaryotic cells to acidify intracellular compartments through the ATP-driven translocation of protons (Nishi and Forgac, 2002; Mindell, 2012). To determine if the V_o_V_1_ ATPase was playing a role in the acid stress response we quantified the transcript levels of the V_o_V_1_ ATPase components (*bb090, bb091, bb092, bb093, bb094, bb096*). We found that all six components of the V_o_V_1_ ATPase had increased expression in response to propionate (**Figure 3A**). The most significant increases were in the V_1_ ATP-binding components (*bb093, bb096*) and the V_o_ proton translocation (*bb091*) component of the V_o_V_1_ ATPase. We also quantified the expression of the arginine deiminase pathway genes (*arcA/bb841-arcB/bb842*-*arcD/bb843)*, whose products can protect bacteria from acid stress through the ArcA-mediated conversion of arginine to citrulline and ammonia (Marquis et al., 1987; Ryan et al., 2009; Cusumano and Caparon, 2015). All of these genes had increased expression levels in response to propionate with statistically significant increases in both *arcB/bb842* and *arcD/bb843* transcript levels (**Figure 3A**).

In response to acid stress, bacteria must maintain both the pH_i_ and their PMF to survive (Bearson et al., 1997; Foster, 1999, 2004; Slonczewski et al., 2009). *B. burgdorferi* encodes several transporters/symporters that could play important roles in maintaining PMF homeostasis under acid stress conditions through the transport of protons/ions or monocarboxylic acids. We found that specific transport systems were induced in response to acid stress. Specifically, a Na/H^+^ transporter (*bb638*), glutamic acid transporter (*bb729*), potassium transporters (*bb724, bb725*), and a magnesium transporter (*bb380*) were all significantly induced in response to propionate (**Figure 3B**). However, the Na^+^, Ca^++^, K^+^ transporter *bb164* and the Na^+^/H^+^ transporter *bb447* remained relatively unchanged (**Figure 3B**). Taken together, these results suggest that in response to acid stress, *B. burgdorferi* initiates an acid stress response that promotes the transcription of select genes encoding proteins that can potentially mitigate the detrimental effects of excess protons in the cytoplasm.

### Arginine Inhibits Acidification of the *B. burgdorferi* Cytoplasm

**FIGURE 4:**
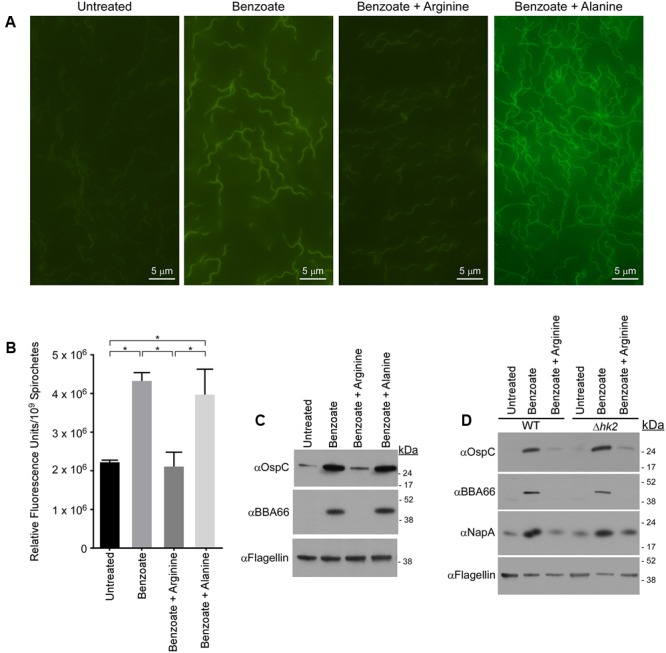
Arginine inhibits acidification of the *B. burgdorferi* cytoplasm and prevents induction of RpoS-regulated proteins independent of the histidine kinase Hk2. **(A)** Fluorescence microscopy or **(B)** quantitative microplate assay of spirochetes grown in media alone (untreated), or in the presence of 20 mM benzoate, benzoate and arginine, or benzoate and alanine and stained with the membrane-permeable pH-sensitive dye pHrodo Green. Increased fluorescence indicates a decrease in pH and the mean value from three biological replicates is shown with the standard deviation. Asterisks denote a statistically significant difference of the mean determined using a one-way ANOVA where *p* < 0.05. **(C)** Immunoblot of OspC and BBA66 in *B. burgdorferi* cells treated with benzoate, benzoate and arginine, or benzoate and alanine. **(D)** Immunoblot of cell lysates from *B. burgdorferi* B31-A3 (WT) and *B. burgdorferi* B3-A3Δ*hk2* (Δ*hk2*) for RpoS-dependent virulence factors OspC, BBA66 and the BosR-regulated factor NapA/BB690 in response to growth in BSK-II or BSK-II supplemented with benzoate, or benzoate and arginine. Flagellin levels were used as a loading control and the position of molecular weight standards is indicated on the right in kDa. With the exception of the microplate assay which was performed in biological duplicate and technical triplicate, all experiments were performed with three biological replicates and representative immunoblots are presented in **(C,D)**. Images for **(A)** were selected at random from images found in Supplementary Figure S2.

We next sought to define conditions that would inhibit acidification of the cytoplasm or inhibit permeable acid-mediated activation of the Rrp2–RpoN–RpoS pathway. To test if arginine could prevent the acidification of the *B. burgdorferi* cytoplasm, either through direct, or indirect buffering through the deamination of arginine and production of ammonia, we again monitored pH_i_ using the pH-sensitive fluorescent dye pHrodo Green. *B. burgdorferi* was grown in media containing benzoate, benzoate and arginine, or benzoate and alanine, stained with pHrodo Green and intracellular fluorescence was evaluated by fluorescence microscopy (**Figure 4A**). Benzoate has been used to study the acid stress response in *E. coli* due to its ability to decrease pH_i_ and induce a robust acid stress response (Wilks and Slonczewski, 2007; Slonczewski et al., 2009), and in *B. burgdorferi* benzoate had the greatest effect on pH_i_ and OspC and BBA66 expression. Therefore, we utilized benzoate to promote acid stress and to make any reduction in fluorescence or protein expression easily detectable in the presence of arginine. Spirochetes grown in BSK-II alone showed weak fluorescence, indicating a near neutral pH (**Figure 4A**, untreated) and spirochetes grown in BSK-II containing benzoate were markedly brighter, suggesting a decreased pH_i_ (**Figure 4A**, benzoate). However, spirochetes grown in both benzoate and arginine had fluorescence levels similar to the untreated controls, suggesting a near neutral pH_i_ (**Figure 4A**, benzoate + arginine). To test whether this buffering effect was specific to arginine, spirochetes were grown in BSK-II containing benzoate and alanine and fluorescence levels were found to be equivalent to the benzoate treated samples (**Figure 4A**, benzoate + alanine). To better characterize the effects of arginine on permeable acid-mediated changes to pH_i_, we next measured pH_i_ changes using pHrodo Green in a quantitative microplate assay. Similar to what we had seen by fluorescence microscopy, spirochetes grown in the presence of benzoate had significantly higher relative fluorescence levels in the microplate assay (**Figure 4B**). Unlike spirochetes grown in benzoate, spirochetes grown in both benzoate and arginine had relative fluorescence levels similar to the untreated controls (**Figure 4B**). The relative fluorescence levels of spirochetes grown in media containing benzoate and alanine were equivalent to the benzoate treated samples (**Figure 4B**). These results indicate that supplementing with arginine can inhibit the acidification of the *B. burgdorferi* cytoplasm that occurs in response to benzoate.

### Activation of the Rrp2–RpoN–RpoS Signaling Cascade Is Dependent on pH_i_ and Independent of the Histidine Kinase Hk2

If the Rrp2–RpoN–RpoS cascade is activated due to a decrease in pH_i_, then the addition of arginine should inhibit induction of the Rrp2–RpoN–RpoS cascade. To test this hypothesis, we grew *B. burgdorferi* in the presence of both benzoate and arginine and monitored OspC and BBA66 levels by immunoblot. To ensure that any affect attributed to arginine was specific to arginine, WT B31-A3 spirochetes were cultured in BSK-II containing benzoate and alanine. Levels of OspC and BBA66 were again increased in response to benzoate, however, not in the presence of both benzoate and arginine (**Figures 4C,D**). The addition of alanine was unable to inhibit the permeable acid-mediated induction of OspC and BBA66 and arginine alone did not promote activation of the Rrp2–RpoN–RpoS cascade (data not shown). Taken together with our previous data, these results suggest that pH_i_ and virulence factor expression are coupled in *B. burgdorferi*. A decrease in pH_i_ can activate the Rrp2–RpoN–RpoS cascade and promote virulence factor expression.

We next focused on the molecular mechanism underlying the activation of the Rrp2–RpoN–RpoS cascade under acid stress. The response regulator Rrp2 is essential in *B. burgdorferi* (Groshong et al., 2012) and its cognate histidine kinase-2 (Hk2) has no known function. We tested whether Hk2 might play a role in activation of the Rrp2–RpoN–RpoS cascade under acid stress. Strains B31-A3 (WT) and B31-A3Δ*hk2*(Δ*hk2*) *B. burgdorferi* (Xu et al., 2010) were grown in BSK-II media supplemented with benzoate or benzoate and arginine and immunoblots of cell lysates demonstrated that OspC and BBA66 were increased in response to benzoate, but not in the presence of benzoate and arginine (**Figure 4D**). Additionally, we analyzed the level of NapA/BB690 levels to determine what affect benzoate and arginine would have, if any, on the oxidative stress response. Similar to what we observed for OspC and BBA66, NapA/BB690 levels increased in the presence of benzoate and levels were similar to the untreated sample in the benzoate and arginine treated samples (**Figure 4D**). These data indicate that Hk2 does not play a role in the permeable acid-mediated induction of RpoS-dependent factors (**Figure 4D**), or in promoting the expression of NapA/BB690. These results are consistent with what has been previously shown for the acetate-triggered induction of RpoS (Xu et al., 2010) and Hk2 does not play a role in the arginine-mediated inhibition of the Rrp2–RpoN–RpoS cascade or in expression of NapA/BB690.

During the infectious cycle, *B. burgdorferi* survives in complex environments that require changes in gene expression to ensure survival and transmission. In response to the weak organic acid propionate, spirochetes initiate an acid stress response, promoting the expression of genes whose products can mitigate the detrimental effects of excess protons in the cytoplasm. Moreover, several monocarboxylic acids trigger the acid stress response that promotes RpoN- and RpoS-dependent virulence factor expression and a portion of the oxidative stress response regulon. It has long been supposed that *B. burgdorferi* does not have typical stress response mechanisms (Caimano et al., 2004); however, these data suggest that the acid stress response and virulence factor expression are likely linked, possibly to promote transmission or to facilitate long-term survival in the tick.

### Lactate and Acetate Are Present in the Tick Midgut Following Feeding

Membrane-permeable acids can promote the expression of RpoS-dependent virulence factors and trigger an acid stress response in *B. burgdorferi*. However, whether membrane-permeable acids are present throughout the infectious cycle is unknown. Although the biochemical composition of some relevant biological tissues where *B. burgdorferi* resides during the infectious cycle is known, the composition of the *I. scapularis* midgut is poorly defined. The membrane-permeable acids acetate, butyrate, and propionate have been identified in the midgut of spirochete-containing arthropods (Odelson and Breznak, 1983; Gijzen and Barugahare, 1992) and bacteria identified in the tick midgut, including *B. burgdorferi*, produce membrane-permeable acids as metabolic end-products (Fraser et al., 1997; Narasimhan et al., 2014). Therefore, to assess the potential role for membrane-permeable acids on signaling during the infectious cycle, we determined the concentration of membrane-permeable acids in the midgut of *I. scapularis* and in relevant biological fluids of host animals.

**FIGURE 5:**
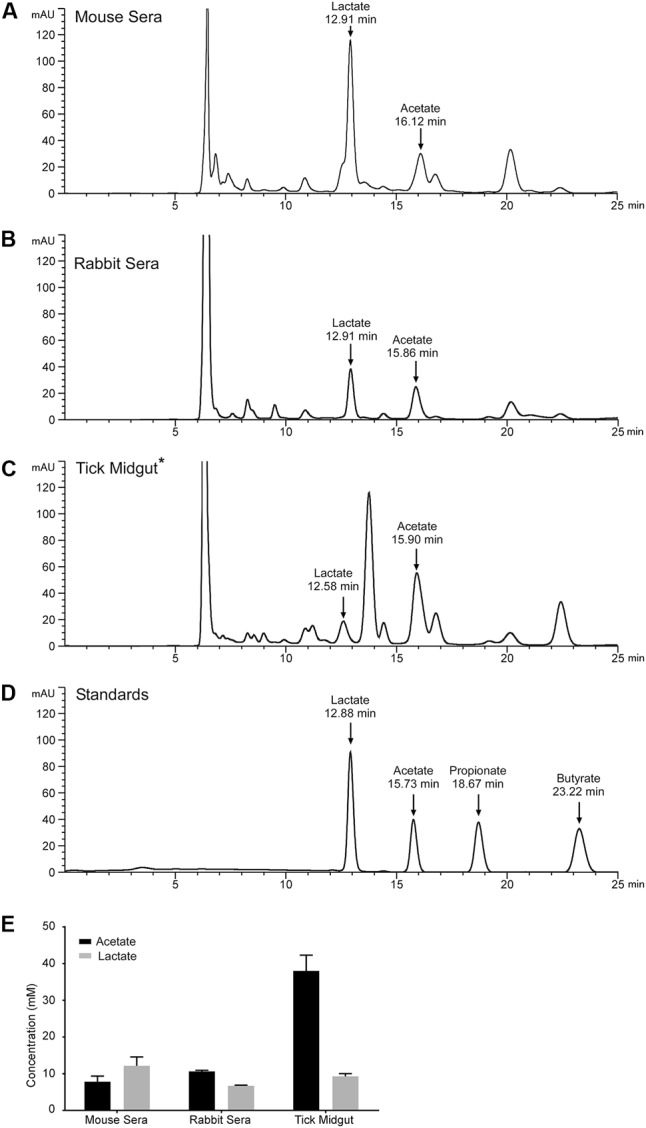
Lactate and acetate are encountered by *B. burgdorferi* during the infectious cycle. Representative HPLC chromatograms monitoring absorbance at 210 nm of samples prepared from **(A)** mouse sera, **(B)** rabbit sera, **(C)** *B. burgdorferi*-infected tick midgut material, or **(D)** a standard containing lactate, acetate, propionate, and butyrate. Arrows indicate peaks confirmed by mass spectroscopy corresponding to lactate, acetate, propionate, and butyrate with their relative retention times listed in minutes. Asterisk indicates that the sample was diluted 1:1 with water prior to being run on the HPLC. Benzoate has a retention time of 30.1 min and is not displayed on this chromatogram. **(E)** Mean concentration of lactate or acetate found in mouse sera, rabbit sera, or the *B. burgdorferi*-infected tick midgut determined by HPLC analysis. Quantification of lactate and acetate was performed in biological triplicate for mouse sera and tick midgut contents. One rabbit blood sample was taken and sample preparation and HPLC/MS analysis was performed in technical triplicate.

We measured the levels of monoprotic acids (lactate, acetate, propionate, butyrate, and benzoate), using reverse phase high-pressure liquid chromatography (HPLC) and mass spectrometry (MS), in mouse sera, rabbit sera, and *I. scapularis* (*B. burgdorferi*-infected) midgut contents for the presence of these acids. Samples were isolated and processed as described previously and monocarboxylic acids were separated by HPLC and detected at 210 nm (**Figures 5A–C**). Peaks corresponding to acetate and lactate were identified based on their relative retention times compared to authentic standards (**Figure 5D**) and their identity was confirmed by MS. Mouse and rabbit sera had significant levels of lactate (∼12.1 ± 2.43 and ∼6.7 ± 0.17 mM, respectively) and acetate (∼7.8 ± 1.53 and ∼10.6 ± 0.3 mM, respectively). These two monoprotic acids were the predominant species detected in these sera samples. Midgut contents harvested from replete, adult *I. scapularis* ticks that had fed upon the same rabbit whose sera we measured had a concentration of lactate (∼9.3 ± 0.8 mM) similar to levels measured in the rabbit sera (**Figure 5E**), while the acetate concentration was significantly higher (38.0 ± 4.3 mM). We were unable to detect butyrate, propionate, or benzoate in any of the samples analyzed.

Initially, we had expected the levels of acetate and lactate to be increased in the tick midgut compared to levels initially detected in rabbit sera. That expectation was based upon the hypothesis that the removal of water from the blood meal, to facilitate saliva production in the feeding tick, would effectively increase the concentration of these acids in the midgut contents. While this might be the case for acetate, it appears that lactate, which is the end-product of *B. burgdorferi* metabolism is not increasing, suggesting that this acid might be consumed by the tick or by the resident midgut microbiota (Narasimhan et al., 2014).

Taken together, these data indicate that: (i) a weak organic acid could trigger a significant acid stress response in *B. burgdorferi* by affecting the intracellular pH of the cells, (ii) *B. burgdorferi* could mitigate these effects by generating or transporting neutralizing compounds (e.g., NH_4_, arginine) or “pumping” protons out of the cells (V_o_V_1_ ATPase), (iii) the acid stress response and virulence factor expression (e.g., OspC) was regulated by RpoN and RpoS, and (iv) acetate was present in the tick midgut at concentrations high enough to promote virulence factor expression via RpoS-dependent gene regulation *in vivo* to possibly promote long-term survival in the tick midgut.

## Discussion

The effects of acid stress have been best characterized in enteric bacteria exposed to drastic changes in extracellular pH as they pass from the gastric stomach into the lower digestive tract. Despite the drastic change in pH due to concentrated HCl, *E. coli* and *Salmonella* rebound very rapidly restoring pH homeostasis. To study the protective mechanisms that these bacteria use to defend against extreme pH changes, it is necessary to use monocarboxylic acids to dramatically extend the time it takes for the cytosol to rebound to pH homeostasis (Slonczewski et al., 2009). Protonated weak acids will diffuse through bacterial cell membranes, where the higher internal pH causes deprotonation that affects pH_i_ and the PMF. The concentration of the protonated acid is a function of the p*K*_a_ of the acid and the pH of the solution. The closer the pH of the solution is to the p*K*_a_ of the acid, the higher the proportion of protonated acid available. Additionally, in well-buffered solutions separated by a lipid bilayer, the rate of diffusion of weak acids across a membrane is increased, as the buffer increases the concentration of protonated acid species available (Gutknecht and Tosteson, 1973). The p*K*_a_ of the acids used in our study suggest that there was a correlation between the proportion of protonated acids available and the level of expression of RpoS and RpoS-dependent factors. We found that the closer the p*K*_a_ of the acid is to the pH of the media (pH 7.0), the greater the increase in RpoS expression (propionate p*K*_a_ = 4.88, acetate p*K*_a_ = 4.76, benzoate p*K*_a_ = 4.2, lactate p*K*_a_ = 3.86) (**Figures 1C, 2B**). However, benzoate did not follow this trend. Due to its non-polar ring structure, benzoate can also diffuse across a membrane in an unprotonated form and can act as an un-coupler of respiratory-chain-generated membrane potential, it is much more difficult for bacterial cells to reestablish pH homeostasis when exposed to this monocarboxylic acid. Moreover, propionate, unlike acetate (used in undecaprenyl-P synthesis) and lactate (used in anaerobic metabolism and secreted by BB0604), is not metabolized or actively transported/exported by *B. burgdorferi*. With no transporters or enzymes to eliminate benzoate or propionate, they likely accumulate in the cytoplasm, resulting in the greatest increase in expression of components of the Rrp2–RpoN–RpoS cascade. Additionally, induction of RpoS and OspC by weak organic acids seems limited to monocarboxylic acids as dicarboxylic acids, like succinate, failed to induce expression of OspC.

Neutralophiles, like *B. burgdorferi*, use a variety of mechanisms to deal with perturbations to intracellular pH. Analysis of the *B. burgdorferi* genome identified genes encoding enzymes that have been previously shown in other bacteria to restore a favorable pH_i_. These include: (i) putative coupled proton exchange with cations (BB0637–BB0638, BB0447, BB0724–BB0725, BB0380) and Na^+^/H^+^ antiporters (BB0637–BB0638); (ii) transporting neutralizing buffering molecules (polyamines: BB0639–BB0640); (iii) amino acid deaminases (BB0841–BB0843); (iv) and proton export via a V_o_V_1_ vacuolar-type ATPase (BB090–BB096). Cationic transport systems are highly conserved among bacteria and have been shown to act as primary proton pumps for restoring pH homeostasis in acidophiles, alkaliphiles as well as neutralophiles (Slonczewski et al., 2009). Na^+^/H^+^ antiporters are also highly conserved among bacteria and are extremely important for restoring pH homeostasis in acidophiles and neutralophiles (Slonczewski et al., 2009). Indeed, these systems have been shown to be induced by a differential in pH_i_ vs pH_e_ and this seemed to be the case for these genes in *B. burgdorferi* (Foster, 2004; Slonczewski et al., 2009; Wilks et al., 2009). The role of the putative glutamate transporters (BB0401 and BB0729) in the acid stress response was difficult to understand since *B. burgdorferi* does not harbor the genes encoding any glutamate or lysine decarboxylases. These are the most common enzymes involved in restoring pH homeostasis in most neutralophiles. Additionally, BB0522 can potentially convert glutamate to glutamine in the presence of NH_3_ suggesting that glutamate may be used to generate glutamine which could act as an effective intracellular buffer. At this time, we do not have any experimental evidence that helps define the role of the glutamate transporters in the acid stress response. The arginine (ArcD/BB0843) transporter was also upregulated when *B. burgdorferi* cells are exposed to propionate and, more importantly, exogenous arginine could neutralize the effects of the strongest, non-metabolizable monocarboxylic acid tested (benzoate). ArcD putatively functions as an arginine/ornithine symport system. After transport, arginine can be hydrolyzed to citrulline + NH_3_ by ArcA and ultimately to ornithine + carbamoyl-P by ArcB/BB0842. Arginine and ornithine would be excellent cytosolic buffering molecules as would NH_3_. As important, NH_3_ which cannot be utilized in amino acid or nucleic acid biosynthetic pathways, could be conjugated to glutamate (forming glutamine) to be incorporated into newly synthesized proteins. The induction of the genes encoding the V_o_V_1_ vacuolar ATPase during acid stress was fascinating and, in retrospect, predictable. Without respiratory enzyme complexes, *B. burgdorferi* utilizes this ATPase to hydrolyze ATP and pump protons across the cytoplasmic membrane to maintain the PMF which is required for motility and energizes critical transport systems. It seemed very likely that the vacuolar ATPase was a major defense against acid/proton stress. It should be noted that the lack of induction of some of the cation transport systems does not mean that they do not play a role in restoring pH homeostasis. It has been shown that constitutive expression of these systems is adequate to protect bacteria from acid stress (Foster, 1999; Slonczewski et al., 2009). What seems clear is that *B. burgdorferi* makes use of a unique combination of enzymes to maintain pH homeostasis that are specifically tailored to the environments in which it survives.

To sense its environment, *B. burgdorferi* harbors the genes encoding only two two-component regulatory systems: Hk1-Rrp1 which control the secondary messenger, cyclic-di-GMP; and Hk2-Rrp2 which, through this RpoN-specific response regulator, controls the transcription of RpoS (Samuels, 2011). Unlike more canonical response regulators, Rrp2 lacks a trans-membrane domain and cannot interact directly with extracellular ligands (Yang et al., 2003). How Rrp2 senses environmental signals and translates those into appropriate changes in gene expression remains to be elucidated. Our data demonstrated that a decrease in pH_i_ promoted the transcription of RpoN- and RpoS-dependent genes, indicating that the Rrp2–RpoN–RpoS signaling cascade was activated under these conditions (Xu et al., 2010; Richards et al., 2015). Additionally, when we neutralized the acidification of the cytoplasm, we prevented the activation of the Rrp2–RpoN–RpoS cascade (**Figures 4A–C**). Changes in intracellular pH can affect multiple cellular functions simultaneously, including enzyme function and disruption of the PMF. In *B. burgdorferi*, with few proton-pumping transporters and lacking respiratory enzymes, the pH_i_ and the PMF are inextricably linked. Therefore, a weak acid mediated decrease in pH_i_ or disruption of the PMF, could be an intracellular signal that reflects changes in the extracellular environment. Moreover, a decrease in pH_i_ or disruption of the PMF likely results in a decrease in the intracellular concentration of ATP as the vacuolar ATPase hydrolyzes ATP to export protons to maintain the PMF and restore pH homeostasis. This decrease in the intracellular energy pool could also be an activation signal for the Rrp2–RpoN–RpoS pathway. Currently, the vacuolar ATPase is thought to be the major contributor to generating the PMF (Radolf and Samuels, 2010), making it difficult to separate the PMF from the vacuolar ATPase activity in *B. burgdorferi*. Further mechanistic studies to determine the function of Rrp2 should help elucidate how perturbations to pH homeostasis activate this pathway.

Whether membrane-permeable acids found in the *I. scapularis* midgut help promote the transmission of *B. burgdorferi* by triggering the expression of virulence factors is unresolved, however, seems unlikely. The monocarboxylic acids acetate and lactate were detected at significant levels late in the feeding cycle, well after the expression of key virulence factors and the initiation of transmission have begun (Schwan and Piesman, 2000). It is more likely that membrane-permeable acids elicit an acid stress response in *B. burgdorferi* to protect spirochetes from multiple stressors found in the tick midgut (e.g., RNS and ROS) and promote long-term survival. The conditions found in the *I. scapularis* midgut, both physiological and biochemical, during and following blood feeding, are likely critical to pathogen transmission and long-term survival.

The tick midgut and its contents are a complex, constantly changing biome in which many conditions, macromolecules and bioactive compounds remain undefined (Yang et al., 2000; Bontemps-Gallo et al., 2016). We now report millimolar concentrations of both lactate and acetate in the midgut of replete ticks (**Figure 5E**). Lactate levels were lower than concentrations measured in rabbit or mouse sera while the concentration of acetate was significantly higher than those measured in animal sera (**Figure 5E**). This result was intriguing since the acetate concentration increased sharply between rabbit sera and the tick midgut contents while lactate levels stayed relatively constant (**Figure 5E**). If the levels of the organic acids detected in the midgut were the result of only the concentration of the blood meal, we would expect lactate and acetate to increase proportionally. Possibly, the observed lactate levels reflect the utilization of this acid by the tick and/or the midgut microbiome at rates exceeding the contribution of the incoming blood or the metabolism of lactate producers like *B. burgdorferi*. In the tick midgut, the concentration of lactate was lower than what we used in our experiments (20 mM lactate vs 9 mM in the midgut) while the acetate concentration was detected in the midgut at a higher concentration than what we used in our experiments (20 mM acetate vs 38 mM in the midgut). Interestingly, even when supplementing 20 mM acetate in the media, RpoS and OspC expression are markedly increased. Furthermore, RpoS and OspC expression increase in a concentration dependent manner when exposed to acetate, with robust expression at acetate concentrations close to those we report in the tick midgut (30 mM acetate vs 35 mM in the midgut) (Xu et al., 2010; Richards et al., 2015).

Currently, we do not know the source of the acetate in the tick midgut and several possible factors may be affecting the acetate concentration we observed. First, these levels may reflect the concentration of a non-utilizable blood component. It seems unlikely that this is the sole explanation considering the dynamic nature of the environment in a feeding tick midgut. Secondly, the midgut microbiota may be contributing acetate generated as a product of microbial metabolism. Microbes present in some arthropods can carry out acetogenesis (H_2_ + CO_2_ → acetate), generating significant amounts of acetate in the gut, which is absorbed by the gut cells and utilized as an energy source (Breznak and Switzer, 1986; Leadbetter et al., 1999). There is no indication that microbes in the tick midgut can perform acetogenesis or that ticks absorb acetate from the midgut. However, the conversion of lactate to acetate seems likely considering the composition of the midgut microflora and that a decrease in the complexity of the tick midgut microbiome has adverse effects on tick physiology (Narasimhan et al., 2014). While these factors may influence the overall observed acetate concentration in the tick midgut, what is clear is that the midgut biochemistry and physiology are complex and we are just beginning to dissect this extremely dynamic environment.

Our study identified an acid stress response in *B. burgdorferi* that activated the Rrp2–RpoN–RpoS signal transduction cascade, some BosR-directed oxidative stress response genes, and increased the expression of genes encoding proteins involved in restoring pH homeostasis. An increase in the transcription of these genes was stimulated by membrane-permeable monocarboxylic acids, some of which (acetate and lactate) were identified in the tick midgut following feeding. Moreover, we have now demonstrated that perturbations in pH homeostasis activated the Rrp2–RpoN–RpoS/BosR regulatory cascade(s) that was previously attributed to Acetyl-P (Xu et al., 2010). We did not identify the intracellular signal that ultimately up regulates these pathways, however, the effects of the decrease in pH_i_ on the vacuolar ATPase might suggest that ATP or GTP may serve as effective intracellular energy “sensors.” While it seemed clear that the acid stress response and pH_i_ effected virulence factor expression in *B. burgdorferi in vitro*, it seemed unlikely that these parameters affect transmission *in vivo*. Since the expression of virulence factors, like OspC, are so tightly linked to RpoS, *in vitro* conditions that trigger Rrp2–RpoN–RpoS and BosR-dependent gene regulation do not necessarily indicate that these conditions are required for, or play a role in, successful transmission. Considering the complexity of the tick midgut, it seems likely that multiple factors are required to maximize and synchronize the expression of virulence factors to the tick feeding cycle to promote successful transmission.

## Materials and Methods

### *Borrelia burgdorferi* Strains and Growth Conditions

*Borrelia burgdorferi* strains B31-A3, B31-A3Δ*rpoS*, B31-A3Δ*rpoN*, B31-A3Δ*bosR*-L9, and B31-A3Δ*hk2* have been described previously (Elias et al., 2000; Fisher et al., 2005; Xu et al., 2010; Katona, 2015). Membrane-permeable acids were tested as previously described (Wilks and Slonczewski, 2007) with minor modifications. *B. burgdorferi* strains were grown at 34°C in BSK-II medium pH 7.0 under microaerobic conditions (3–5% O_2_) to mid-logarithmic phase (5–7 × 10^7^/mL). Spirochetes were subsequently diluted to 1 × 10^6^/mL in BSK-II medium pH 7.0 or BSK-II medium pH 7.0 supplemented with 20 mM sodium acetate, sodium benzoate, sodium lactate, sodium propionate or in combination with 20 mM L-arginine or L-alanine (Sigma-Aldrich, St. Louis, MO, United States).

### SDS-PAGE and Immunoblotting

Spirochetes were grown to 1–5 × 10^7^/mL, harvested by centrifugation, washed twice with HEPES–NaCl buffer (20 mM HEPES, pH 8.0; 50 mM NaCl) (HN) and whole cell lysates were analyzed by SDS-PAGE and immunoblots as previously described (Dulebohn et al., 2014). Antibodies were used at the following concentrations: αOspC 1:1000 (Tilly et al., 2007), αBBA66 1:4000 (Clifton et al., 2006), αNapA/BB690 1:5000 (Boylan et al., 2003), αRpoS 1:500 (Dulebohn et al., 2014), αFlagellin 1:100 (Barbour et al., 1986), and αFur/BosR 1:1000 (Boylan et al., 2003). Immunoblots were incubated in 1× TBS-T with an HRP (horseradish peroxidase) conjugated recombinant protein A (Thermo Fisher Scientific, Grand Island, NY, United States) at 1:4000 for 1 h at RT with shaking for the detection of OspC, NapA/BB690, RpoS, and BosR. For detection of flagellin, an anti-mouse secondary antibody-HRP conjugated antibody was used at 1:10,000 (Thermo Fisher Scientific) and detection of BBA66 was performed with a secondary HRP conjugated αChicken antibody at 1:50,000 in 1× TBS-T as previously described (Clifton et al., 2006). Immunoblots were developed using SuperSignal West Pico chemiluminescent substrate kit (Thermo Fisher Scientific) and X-ray film (Lab Scientific Inc., Livingston, NJ, United States). Quantification of OspC and BBA66 (Supplementary Figure S1) was performed using Image J software (Schneider et al., 2012).

### Quantitative Reverse Transcriptase PCR

qRT-PCR was performed as previously described (Dulebohn et al., 2014) with minor modifications. Briefly, spirochetes were grown in BSK-II medium with or without benzoate or propionate, harvested at 1–5 × 10^7^/mL, and washed with HN buffer. Spirochetes were resuspended in 1 mL of TRI reagent (Sigma-Aldrich) and RNA harvested using a Direct-zol RNA mini prep kit (ZYMO Research, Irvine, CA, United States). RNA (5 μg) was treated for 1 h with Turbo DNase (Thermo Fisher Scientific) and 1 ng subsequently used for cDNA synthesis using High-Capacity cDNA Reverse Transcription Kit (Thermo Fisher Scientific). cDNA was then used in qPCR reactions using the 5′ nuclease Prime Time Assay from IDT on a Viia 7 instrument (Thermo Fisher Scientific). Primers and probes are listed in Supplementary Table S1 and statistical analysis done with GraphPad PRISM software (GraphPad Software Inc., La Jolla, CA, United States).

### β-Galactosidase Activity Assays

Analysis of β-galactosidase activity was performed as previously described (Dulebohn et al., 2014). Briefly, *B. burgdorferi* strain B31-A3 containing the pBH*ospC*p-*lacZ*_Bb_ (Hayes et al., 2010) or pBSV2G-*rpoS*p_92_-*lacZ*_Bb_ shuttle vector, harboring the *ospC* or *rpoS* promoter, respectively, were grown to 1–5 × 10^7^ spirochetes/mL as stated above and β-galactosidase activity was assayed in three biological replicates as previously described (Dulebohn et al., 2014). The pBSV2G-*rpoS*p_92_-*lacZ*_Bb_ shuttle vector was constructed by amplifying the *rpoS* promoter from *B. burgdorferi* B31-A3 gDNA using primers *rpoS*P92-F and *rpoS*P-R (Supplementary Table S1) and the 92 bp fragment (*rpoS*p_92_) was ligated immediately upstream of the *lacZ*_Bb_ gene in pBH*lacZ*_Bb_ (Hayes et al., 2010). Relevant sequences were confirmed by sequencing.

### Cytoplasmic pH Analysis with Membrane-Permeable pH Sensitive Dye

*Borrelia burgdorferi* strain B31-A3 was grown to 1–2 × 10^7^ spirochetes per mL in BSK-II (pH 7.0) and cultures were split and treated with either sodium acetate, sodium lactate, sodium propionate, sodium benzoate or a combination of sodium benzoate and arginine, or sodium benzoate and alanine (20 mM final concentration of each). Cultures were then incubated at 35°C for 48 h under microaerobic conditions (3–5% O_2_) and equivalent numbers of spirochetes (3 × 10^9^ spirochetes) were harvested by centrifugation at 3,000 rpm for 10 min and washed twice with Live Cell Imaging Solution (LCIS) (Thermo Fisher Scientific). pHrodo Green AM (Thermo Fisher Scientific) was then used according to the manufacturer’s instructions, samples incubated at 37°C for 30 min and then washed twice with LCIS. Fluorescence was quantified using a BioTek synergy spectrophotometer (BioTek, Winooski, VT, United States) using Excitation/Emission filters of 485 nm/560 nm, respectively. At least two independent biological replicates were performed for each condition tested and each biological replicate was analyzed in duplicate. Extended imaging led to increased background fluorescence so ReadyProbes^TM^ Backdrop^®^ Green Background Suppressor reagent (Thermo Fisher Scientific) was added to each sample and then imaged on a Nikon Eclipse E80 epifluorescence microscope (100× magnification, CY3 filter). All images were captured with identical exposure times (1 s).

### Analysis of Biological Samples by High-Pressure Liquid Chromatography and Mass Spectrometry

Mouse infection and tick feeding were performed as previously described (Bontemps-Gallo et al., 2016). Briefly, laboratory-raised *I. scapularis* larvae (Oklahoma State University) were fed to repletion on *B. burgdorferi* B31-A3 infected mice, collected and allowed to molt to nymphs. Nymphs were then fed to repletion on naïve RML mice, collected and allowed to molt into adults. Adult ticks were then fed to repletion on New Zealand White rabbits. Midgut material was collected as previously described (Bontemps-Gallo et al., 2016) and diluted 1:2 in sterile water. Blood samples from New Zealand White rabbits or RML mice were clarified by centrifugation at 2000 × *g* for 10 min and the serum fraction recovered. All samples were cleared by centrifugation using a Microcon-10 (EMD Millipore, Billerica, MA, United States) at 10,000 × *g* until completion. The filtrate was then analyzed using an Agilent 1200 Series liquid chromatography system coupled to a 6120 Series Single Quad mass spectrometer equipped with an ESI source (Agilent Technologies, Santa Clara, CA, United States). Twenty microliters of sample or standard was injected and separated on an Aminex HPX-87 300 mm × 7.8 mm column (Bio-Rad, Hercules, CA, United States) following the manufacturers protocol. Absorbance data was collected from 200 to 750 nm to monitor COOH groups at 210 nm and aid in compound identification and peak purity analysis. Negative ion mass data was acquired as previously described (Ibanez and Bauer, 2014), with a 350°C nitrogen gas temperature and select ion monitoring for the [M-H]^-^ of acetic acid (59.00), lactic acid (89.1), propionic acid (73.1), and butyric acid (88.1). The mass spectrometer was tuned to the following internal mass reference ions (*m*/*z*): 112.99, 601.98, 1033.99, and 1633.95. Sample identifications were made by retention time relative to a standard and confirmed by MS. Quantification of lactate and acetate was performed with biological triplicates for mouse sera and tick midgut contents. One rabbit blood sample was taken and sample preparation and HPLC/MS analysis was performed in triplicate. Quantification of lactate and acetate was performed by monitoring UV absorbance at 210 nm and calculating the area under the peak relative to a standard curve for each compound.

## Ethics Statement

Mouse infection studies were carried out in accordance with the Animal Welfare Act (AWA 1990), the guidelines of the National Institutes of Health, Public Health Service Policy on Humane Care (PHS 2002) and Use of Laboratory Animals and the United States Institute of Laboratory Animal Resources, National Research Council, Guide for the Care and Use of Laboratory Animals. All animal work was done according to protocols approved by the Rocky Mountain Laboratories, NIAID, NIH Animal Care and Use Committee (Protocol Number 2014–021). The Rocky Mountain Laboratories are accredited by the International Association for Assessment and Accreditation of Laboratory Animal Care (AAALAC).

## Author Contributions

DD designed and performed the experiments, analyzed the data, and wrote the manuscript; CR, HS, and KL designed and performed the experiments; FG conceived the project, provided reagents and conceptual design, and wrote the manuscript.

## Conflict of Interest Statement

The authors declare that the research was conducted in the absence of any commercial or financial relationships that could be construed as a potential conflict of interest.
