# Supplementary material for: Weak Organic Acids Decrease Borrelia burgdorferi Cytoplasmic pH, Eliciting an Acid Stress Response and Impacting RpoN- and RpoS-Dependent Gene Expression
Source: Front Microbiol. 2017 Sep 29;8:1734. doi: 10.3389/fmicb.2017.01734 (PMC5626856; doi:10.3389/fmicb.2017.01734)
Supplement: Supplementary file 1 [file Table_1.DOCX]

Supplementary Table 1.

Primers and probes used for qRT-PCR

| **Gene** | **Gene Product** | **Forward Primer (5'->3')** | **Reverse Primer (5'->3')** | **Probe*** |
| --- | --- | --- | --- | --- |
| *ospC* | RpoS-dependent Virulence Factor Gene | TCAGGGAAAGATGGGAATACATC | ACAGCAAGT AAAACCGCA TTAG | AGGCCCTTTAACAGACTCATCAGCAG |
| *bba66* | RpoS-dependent Virulence Factor Gene | GTTACAACCGTACCCGGAAATA | GCTGTCTTG TTGGTTGAC TAAAG | CGGGCTGCCAACATTTGCCTTAAA |
| *napA/*  *bb690* | BosR-dependent factor | ACTGCTGGTGATTATGGT ACTG | TCGCAACAT TTGCTTTCA TTCT | TGGATGCATAAGGCATTGCTTGAA |
| *bba34* | RpoS-dependent Virulence Factor Gene | TCTTACGACAACACCTGC TATAC | CTCTAATGG TGCTTGCTG TTATTT | AGAGACGACATTTATTGGAGTGACGGC |
| *bbj24* | RpoS-dependent Virulence Factor Gene | AGATGATAATGTTCCAGC GGATTA | TCCTTTCCTATTGTGCGGTATT | TTTAGGGCATTGGGAGAGCTTGCT |
| *bb0728/cdr* | CoADR/Co-A Reductase | CTGCAGGAGATTGTGCAACTA | TGCTGTATG ATTCCCAGC TAAA | CCCTTGGCAACAACAGCCAACAAA |
| *bb0637* | Na^+^/H^+^ pump | ATTGAAGTAAGAGGG CAGCC | AAAACATTG CAACACTAGCCG | AACTCCCAAATAAATCCCCGTGCCT |
| *bb0638* | Na^+^/H^+^ pump | GGGTTTGATTGGTTGCTCTT | CCACTCATT AAAATAGCAAGCCC | TGCAAGAAACATGGTCAAAATGCCAACT |
| *bb0729* | Hypothetical Glutamic Acid Symporter | CCCCACAGATATTTCATT TATACTCAC | CAAGTCCTA CCAATCCCA CTG | CGCAACAACAGCCTCACTAATGGT |
| *bb0841/arcA* | ArcA - Arginine Deiminase | CTCGCTAGACGGAATTCT GTG | GGTGCA AGAGAA CAATGGAAT G | CCTGGAGCTATCGCCAAAACATTAGCA |
| *bb0842/arcB* | ArcB - Ornithine Carbamoyltransferase | TCTAAGGGTAATCAA | CGCCAAACATTCAACAGT CTG | CCGCTAGAGTTTTGGGACGCATGTA |
| *bb0843/arcD* | Arginine/  Ornithine Antiporter | CAGTAGAAGCCATAGTTCCCAC | CGGAATGAGTGAAGAGAC CC | CCGCTCCAACAAGACTATCATAACCCA |
| *rrp2* | Response Regulator-2 | ATTCTAACAGCCCACGGA AC | AAGTCTTTC AAGGTCTAA GGGC | ACCCTCTCTCATGGCATCTACAGCA |
| *rpoN* | RNA Polymerase Alternative Sigma Factor-RpoN | GCAAAGAATTTAACACGGCTTTAG | AATTAGTGT CGTTTGGGT CTTT | CCCAACCCAACGCTCGAATTT |
| *rpoS* | RNA Polymerase Alternative Sigma Factor- RpoS | CACAAGCTAATTACTCAC GAAGAAG | AGTCGCAAGTTTGCATTTATCA | TGCATTGCCTCTTTGTATTTGTCCTGC |
| *bosR* | Borrelia Oxidative Stress Response Regulator- BosR | TAGACGTACATTCCGCAT TGG | CTGTTTCTCGAATGAGATGCTTTC | AGTCGGCATTACAAACGATCCTGT |
| *eno* | Enolase | GTGCACACTCTGACAACT CT | ACCTCTGCT GCCATTCTT ATT | CAGGAGTTCATGATAATGCCAATAGGAGCA |
| *bb0090* | V_0_V_1_ ATPase Component | CTGCTATTGGAGCATGGAAGA | GAGTCAATG GTGCTGAAA CAAA | ATGCAAGGAAAGCCAGCACCATTT |
| *bb0091* | V_0_V_1_ ATPase Component | CCAATTTAGCTGCACCCA TTT | TCCATAAGC AGCATCACC TATT | AGAGACAATTCCTGGGTACAAAGAAAGGG |
| *bb0092* | V_0_V_1_ ATPase Component | TGATACCAACAGCTA AGGCTAATA | GCAATCTTA CCTCTTACA ACAGC | GCAATCTTACCTCTTACAACAGC |
| *bb0093* | V_0_V_1_ ATPase Component | GGCCCTTCTCTTGATGATAATTTG | ATTGGAAGC CCTGTCCTT ATC | TTGGTGGGCCTTCTGCAAATCCTA |
| *bb0094* | V_0_V_1_ ATPase Component | TTGCATTCCGGGTCCTTT | CTCACCACA AGCTGCAAT AATC | ACGGTTCTTCAGCAGGTTACAAGTCG |
| *bb0096* | V_0_V_1_ ATPase Component | GAAGAAGCTGAGAGAGCATCTAAT | TTCAAGGGC ATGGCATTTATAATC | AGTTGCTAGAGCAGAAGAAGCTGCT |
| *bb0380* | *mgtE* (Mg++ transporter) | CTCAGGCATCTGCGCTAATAA | TTGCTCCCA CTAGAATGCTAAC | AGCTTGCTCTTGGTACTGTCAAGGT |
| *bb724* | *ktrA* K^+^ Transport | CCTGAAGCTATAGTTGTG GTCTC | ATCTTGGCATGAAGCGATAGT | TGGTGGGCTTGGGTTTATGGTCT |
| *bb725* | *ktrB* K+ transport | GCTGAAACTGTTATTCCC AAAGA | TCGCCATCA ACAAATTCA TACC | TGAAGCCGATCTTAGAAGAGAATGTGGG |
| *bb0401* | Hypothetical Glutamic Acid transporter | CATCTAGCAATCCAAATCTACTAAGC | ACAATAAAC CCATTTGCA TGGT | AGAGAACTGATGCTAAGCGCATCCA |
|  |  |  |  |  |
| Primers used for pBSV2G-*rpoS*p92-*lacZ*Bb |  |  |  |  |
| *rpoS*P92-F |  | AAGCGGATCCCCATTTTTAAATTAAATTGGCAC |  |  |
| *rpoS*P-R |  | CCGCTCGAGTATTATATTTTCTCCCCTT |  |  |

* Contains a 5' flourophore (6-FAM), an internal ZEN and 3'Iowa Black FQ quencher
