# Supplementary material for: Weak Organic Acids Decrease Borrelia burgdorferi Cytoplasmic pH, Eliciting an Acid Stress Response and Impacting RpoN- and RpoS-Dependent Gene Expression
Source: Front Microbiol. 2017 Sep 29;8:1734. doi: 10.3389/fmicb.2017.01734 (PMC5626856; doi:10.3389/fmicb.2017.01734)
Supplement: Supplementary file 2 [file Image_1.pdf]

Fig S1.

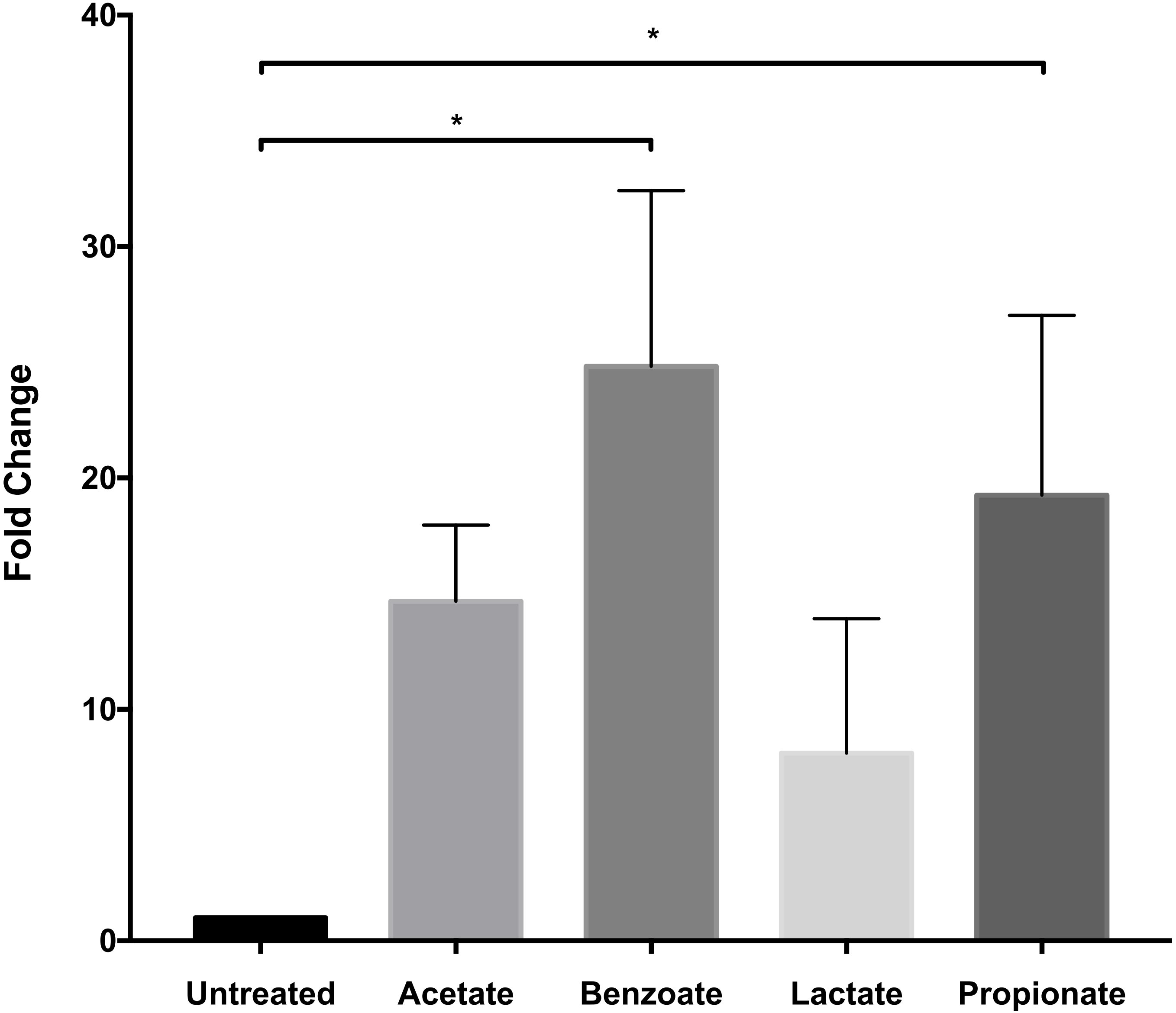

40
30
20
10
0
Fold Change
*
*
Untreated
Acetate
Benzoate
Lactate
Propionate

Fig S2.

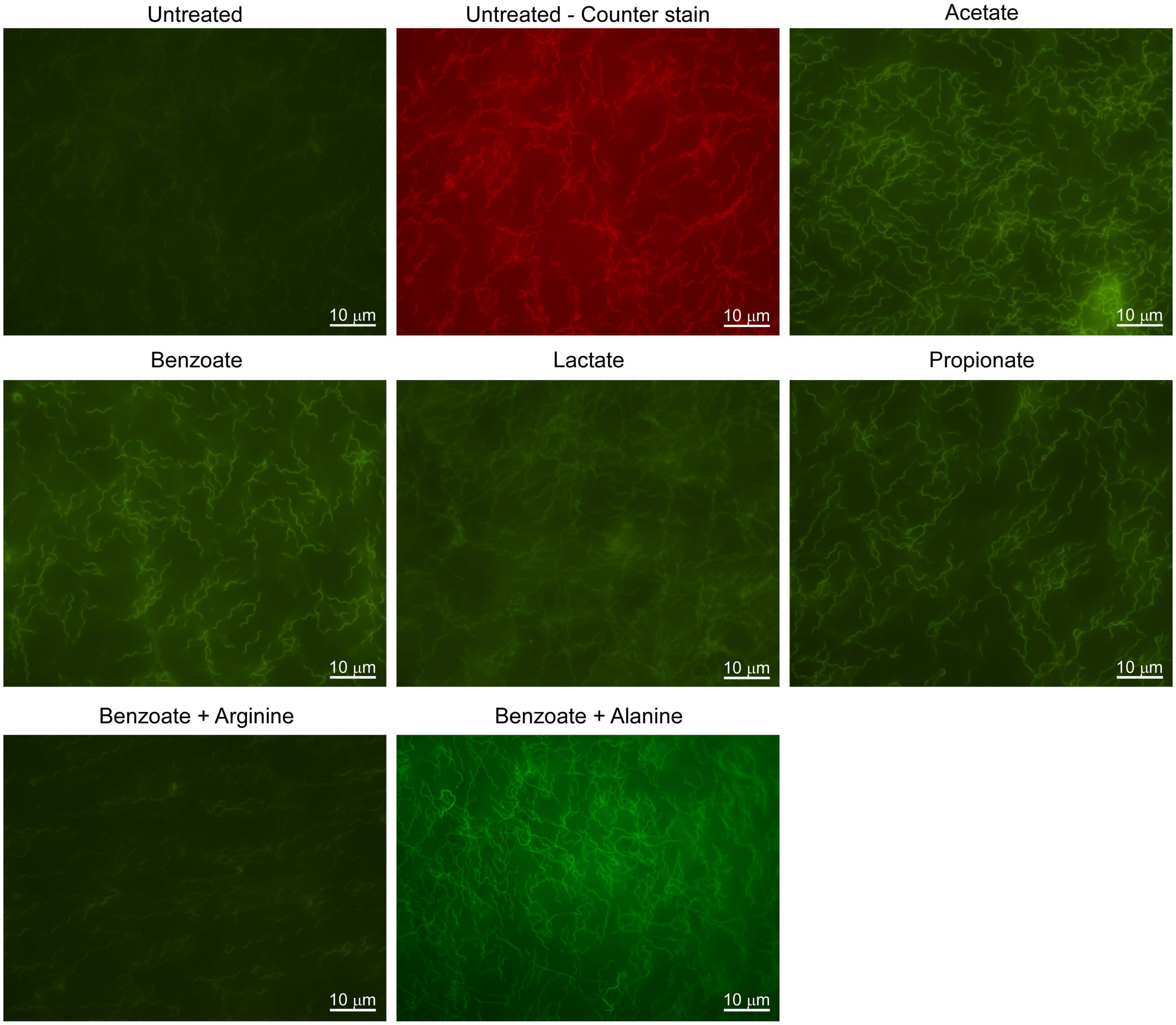
Untreated
Untreated - Counter stain
Acetate
Benzoate
Lactate
Propionate
Benzoate + Arginine
Benzoate + Alanine
10 µm

Fig S3.

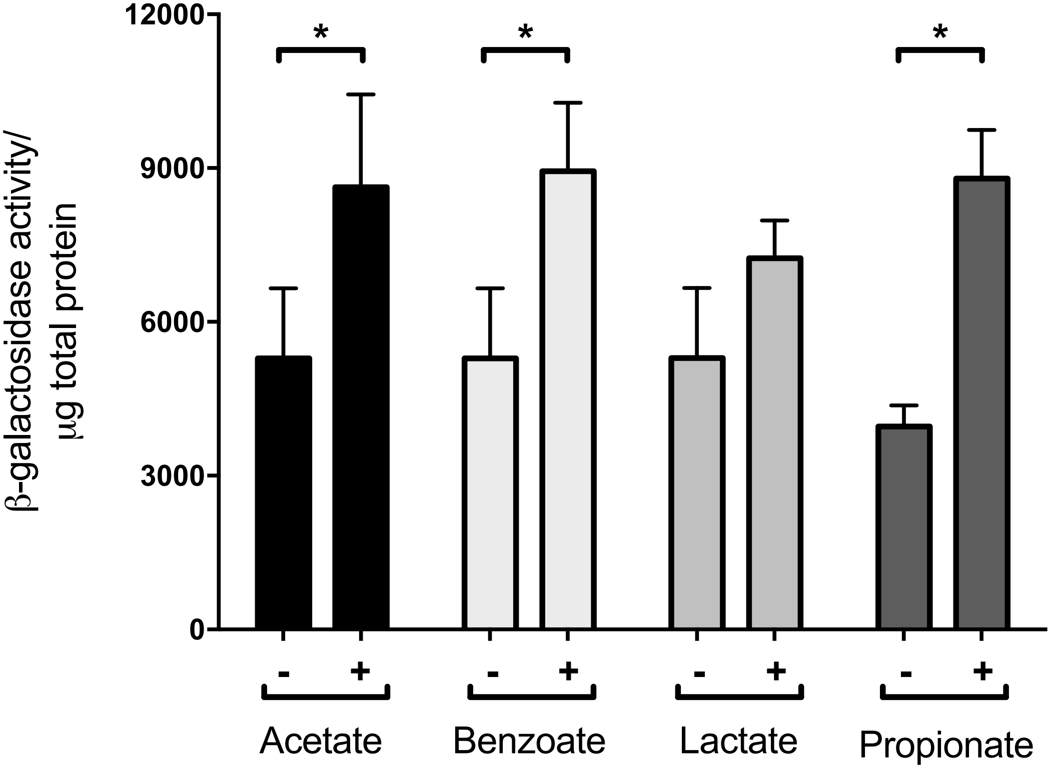

12000
9000
6000
3000
0
*
*
*
β-galactosidase activity/
μg total protein
-
+
-
+
-
+
-
+
Acetate
Benzoate
Lactate
Propionate

Fig S1. Relative fold change in OspC and BBA66 levels

Quantification of immunoblots evaluating the relative fold change of OspC and BBA66 following treatment with acetate, benzoate, lactate or propionate. Immunoblots were quantified using Image J software and a value of "1" was assigned to the untreated control to determine relative fold change. Mean and standard deviation are displayed and asterisks denote a statistically significant difference of the mean determined using a one-way ANOVA where $p<0.05$.

Fig S2. Fluorescence microscopy of *B. burgdorferi*

Fluorescence microscopy of *B. burgdorferi* strain B31-A3 grown in media alone (Untreated) or in the presence of acetate, benzoate, lactate, propionate, benzoate and arginine or benzoate and alanine and stained with the membrane-permeable pH-sensitive dye pHrodo Green. Spirochetes grown in media alone were also stained with a FITC stain to visualize spirochetes (Untreated – Counter stain).

Fig S3. Membrane-permeable acids activate transcription of *lacZ* from the *ospC* promoter.

B. burgdorferi strain B31-A3 harboring a shuttle vector containing a *lacZ* reporter construct was grown in the (-) absence or (+) presence of acetate, benzoate, lactate or propionate and β-galactosidase activity in cell lysates was analyzed. Mean and standard deviation are displayed and asterisks denote a statistically significant difference of the mean determined using a one-way ANOVA where $p<0.05$.
